# Compliance in Patients with Paranoid Schizophrenia and Substance Dependence

**DOI:** 10.1192/j.eurpsy.2025.2043

**Published:** 2025-08-26

**Authors:** M. S. Artemieva, A. T. M. Mkrtchyan, V. P. Sokolov, I. E. Danilin

**Affiliations:** 1 Psychiatry and medical psychology, RUDN University, Moscow, Russian Federation

## Abstract

**Introduction:**

Schizophrenia is one of the most disabling psychiatric disorders, with about 60% of patients also suffering from substance dependence—a rate significantly higher than in the general population. Mentally ill individuals have a suicide risk four times higher than healthy individuals, which doubles when comorbid mental disorders are present. Compliance with treatment in patients with schizophrenia is generally lower than in those with other psychiatric disorders, often due to a lack of continuity between psychiatric and addiction services.

**Objectives:**

This study aims to assess compliance in patients diagnosed with paranoid schizophrenia and substance dependence syndrome and compare it with compliance in patients diagnosed with paranoid schizophrenia without dependence.

**Methods:**

The study included two groups: 15 patients with paranoid schizophrenia and 20 patients with paranoid schizophrenia and substance dependence. The average hospital stay for patients without substance dependence was 25.8 days, whereas it was 38.4 days for those with dependence.

**Results:**

Prolonged hospitalizations increase the economic burden on healthcare and introduce additional challenges, such as job loss, which heightens stigma and marginalization. The number of hospitalizations was also higher among patients with dependence, averaging 4.75 times over five years compared to 1.06 times in those without. Patients without dependence can often remain functional in society on monotherapy, requiring only one medication—a more convenient regimen. In contrast, patients with dependence typically require a combination of three or more medications, with a less flexible and more demanding dosage schedule. These regimens not only increase economic strain but also can worsen medication tolerance. This increases the risk of selective intake, reduced frequency, or complete discontinuation of medications, which often leads to rehospitalization. Frequent therapy adjustments may further erode patients’ adherence to new regimens, undermining their trust in the need to engage with psychiatric care.

**Conclusions:**

As shown, compliance in patients with a “dual diagnosis” is a pressing issue in modern psychiatry. Addressing this complex problem requires multiple steps, including selecting appropriate therapies, addiction treatment, psychoeducation, and fostering a strong doctor-patient relationship in an outpatient setting. These measures collectively aim to reintegrate patients into society, reduce disease burden, improve quality of life, lower suicide risk, and decrease the frequency and length of hospitalizations.

**Disclosure of Interest:**

None Declared

